# Effects of phytonutrient-supplemented diets on the intestinal microbiota of *Cyprinus carpio*

**DOI:** 10.1371/journal.pone.0248537

**Published:** 2021-04-22

**Authors:** Milan Feher, Peter Fauszt, Emese Tolnai, Gabor Fidler, Georgina Pesti-Asboth, Aniko Stagel, Istvan Szucs, Sandor Biro, Judit Remenyik, Melinda Paholcsek, Laszlo Stundl

**Affiliations:** 1 Institute of Animal Husbandry, Faculty of Agricultural and Food Sciences and Environmental Management, University of Debrecen, Debrecen, Hungary; 2 Department of Human Genetics, Faculty of Medicine, University of Debrecen, Debrecen, Hungary; 3 Institute of Food Technology, Faculty of Agricultural and Food Sciences and Environmental Management, University of Debrecen, Debrecen, Hungary; 4 Institute of Applied Economics, Faculty of Economics and Business, University of Debrecen, Debrecen, Hungary; Universidade Catolica Portuguesa, PORTUGAL

## Abstract

In the aquaculture sector, a strategy for the more efficient use of resources and proper disease control is needed to overcome the challenges of meat production worldwide. Modulation of the gastrointestinal tract microbiota is a promising approach for promoting animal health and preventing infection. This feeding experiment was conducted to discover the phytonutrient-induced changes in the gastrointestinal tract microbiota of common carp (*Cyprinus carpio*). Acclimatized animals aged 7 months (30 weeks) were divided randomly into five experimental groups to investigate the effects of the applied feed additives. The dietary supplements were manufactured from anthocyanin-containing processing wastes from the food industry, specifically the production of Hungarian sour cherry extract, synbiotics from fermented corn, and fermentable oligosaccharides from Hungarian sweet red pepper seeds and carotenoids from Hungarian sweet red pepper pulps, applied at a dose of 1%. The gut contents of the animals were collected at four time points throughout the 6-week study period. To track the compositional and diversity changes in the microbiota of the carp intestinal tract, V3-V4 16S rRNA gene-based metagenomic sequencing was performed. The growth performance of common carp juveniles was not significantly affected by supplementation of the basal diet with plant extracts. Phytonutrients improve the community diversity, increase the *Clostridium* and *Lactobacillus* abundances and decrease the abundances of potentially pathogenic and spoilage bacteria, such as *Shewanella*, *Pseudomonas*, *Acinetobacter* and *Aeromonas*. The phyla *Proteobacteria*, *Tenericutes* and *Chlamydiae* were positively correlated with the body weight, whereas *Spirochaetes* and *Firmicutes* exhibited negatively correlations with the body weight. We hypothesize that the application of phytonutrients in aquaculture settings might be a reasonable green approach for easing the usage of antibiotics.

## Introduction

As the human population is expanding, fish have become an nutrition source of increasing importance [1]. The production of total edible aquatic animal food exhibits a greater annual increase than the total terrestrial meat production [2]. The enormously developing aquaculture sector has become the primary source of fish protein and is expected to further expand in order to address the growing needs of the world’s population [3].

The intensification of production has been linked to depressed immunity and decreased resilience to pathogens. Fish diseases have become more significant and lead to serious economic losses [4, 5]. In addition, the lack of proper infection control and limited treatment options negatively affect animal development.

Common carp (*Cyprinus carpio*) is one of the most important freshwater fish species in aquaculture, with a global production of more than 4 million tonnes, which accounts for 7.7% of total finfish production [6]. Additionally, common carp can be produced in both extensive polyculture fish pond systems and intensive monocultural recirculation aquacultures [7–9], which explains its high economic significance [10–13]. Common carp can consume a wide range of different natural foods and is a flexible and opportunistic feeder that can switch from its preferred to alternative diets according to food availability.

The gastrointestinal tract (GIT) microbiota is a diverse population of microorganisms that play various important roles in the biology of multicellular hosts. The genetics, age, physiological status, pathology, metabolic activity, water chemistry, temperature, locations, and trophic level of a specific organism can notably influence the intestinal microbiota of fish [12, 14]. Due to advances in high-throughput sequencing techniques, metagenomes are receiving increased attention, but few studies have investigated fish-associated microbiomes.

One of the key roles of the GIT microbiota is affecting host immunity and nutrition via multiple mechanisms. GIT microorganisms complement digestive processes by providing enzymes that are not encoded by the host’s genome and play important roles in the breakdown of polysaccharides and the synthesis of vitamins [15]. The GIT microbiome also plays a crucial role in colonization prevention and in defence against pathogenic microorganisms by competing for nutrients and adhesion sites and producing antimicrobial substances [15, 16]. Preserving the balance of the GIT microbiota is crucial for maintenance of the intestinal health of fish [17].

Microbial dysbiosis can be triggered by numerous factors leading to either the depletion of beneficial bacteria or the expansion of potential pathogens. By referring to the opening lines of Leo Tolstoy, “all happy families are all alike; each unhappy family is unhappy in its own way”, the Anna Karenina principle can be used to draw a parallel between microbiome-associated diseases and healthy or sick microbiomes: “healthy” microbiomes are alike, and each disease-associated microbiome is “sick” in its own way [18]. One of the key factors in effective disease control is the timely diagnosis of microbial dysbiosis to mitigate devastating outcomes. Increasing our knowledge of the structure of the healthy GIT community, which is known as the symbiome, can aid the recognition of significant shifts in the abundance of certain microorganisms that indicate dysbiosis in aquaculture systems.

Antibiotics and other chemotherapeutics are still indispensable for the control of infections [19], but their wide and excessive use can cause irreversible damage [20–23]. Furthermore, the fish metabolism does not inactivate drugs, and as a result, the pollution of our ecosystems with antibiotic residues has become a major global concern [23]. In particular, many aquatic ecosystems have been destroyed or severely degraded [23].

Bacterial fermentation involves anaerobic carbohydrate metabolism, which converts indigestible dietary carbohydrates into short-chain fatty acids (SCFAs) that can modulate immune responses, disease resistance and energy homoeostasis [24]. In aquaculture, SCFAs and their salts have been used as growth promoters and immune stimulators [25, 26]. The fermentation of SCFAs is related to specific bacteria, but dietary intake has a significant influence on the formation of SCFAs [27].

The application of natural, bioactive components in aquacultures is a fast-growing area. Herbal medicines have recently become the focus of the meat industry by supplying consumers with clean-labelled products free of artificial ingredients or synthetic chemicals.

The use of bacteria for probiotic purposes is a new approach for improving fish health and nutrition. Furthermore, dietary probiotics are known to stimulate nutrient digestion and release vitamins and amino acids to the host. Prebiotics are capable of selectively promoting the growth of beneficial microbiota in the gut of aquatic animals, such as common carp [28–30]. A previous study showed that fermentable oligosaccharides exert positive effects on the bacterial microbiome of aquatic animals [31].

Aquaculturists are interested in natural, bioactive components that might reduce the risk of infections. A growing body of evidence related to improving the gut and overall health of animals indicates that pro- and prebiotics have been widely and successfully used as feed additives to prevent and control infectious diseases in fish [5, 22]. However, further research is needed to determine their optimal dosage and long-term effects [5].

Sour cherry (*Prunus cerasus*) and sweet red pepper (*Capsicum annuum*), which are natively grown plants in Hungary, are famous worldwide for being rich in antioxidants, vitamins and flavonoids and thus exerting several beneficial health effects [32–35].

In this feeding experiment, we investigated the effects of a phytonutrient-enriched diet on the microbiota of the *Cyprinus carpio* GIT. To this end, waste materials from the food industry that are rich in natural bioactive compounds, specifically the waste from the processing of sour cherry (rich in anthocyanins) and sweet red pepper pulp (rich in carotenoids) and seed (rich in fermentable-oligosaccharides), were recycled. We believe that this green and cost-effective approach has exciting potential and might lead to improvements in intensive aquaculture farming.

## Materials and methods

### Experimental protocol

**Fig 1:**
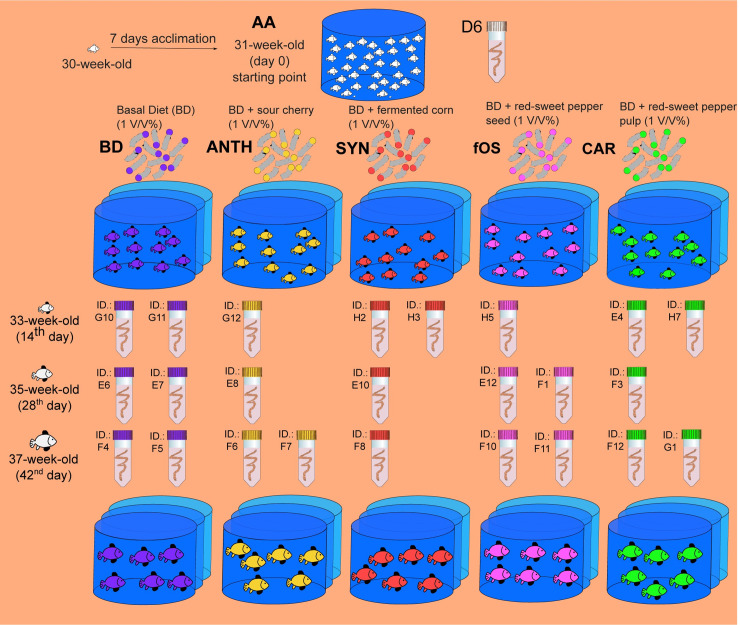
Overview of the study protocol showing the feeding and sampling strategies. AA stands for acclimatized animals aged 7 months (30 weeks). The fish were divided randomly into five experimental groups and fed either the commercial basal diet (BD, negative control) with no dietary supplement or the following dietary treatments as supplemented feed: BD+1% anthocyanins (ANTH) provided by Hungarian sour cherry extract, BD+1% synbiotics (SYN) provided by fermented corn, BD+1% fermentable oligosaccharides (fOS) provided by Hungarian sweet red pepper seed extract, and BD+1% carotenoids (CAR) provided by Hungarian sweet red pepper pulp extract. The fish intestines were collected on the 1^st^, 14^th^, 28^th^ and 42^nd^ days of the experimental period. The letter and number codes (D6-H7) refer to the code used during the 16S metagenomic sequencing library preparation.

The study was approved and performed in accordance with the guidelines of local ethics committee at the University of Debrecen (University of Debrecen Committee of Animal Welfare) under the registration number DEMAB/15/2019. A schematic view of the feeding trial is presented in Fig 1. Common carp juveniles aged 7 months (30 weeks) were used in this pilot study. Fish with an initial body weight of 123.45±0.37 g were obtained through artificial propagation and reared in a water recirculation system prior to the experiment. A total of 165 fish were equally distributed into five treatments groups 1 week before initiation of the feeding test for acclimation (acclimated animals; AA). After 7 days of acclimatization, the AA animals were further randomly sorted into five treatment groups, and the fish belonging to one of three replicates of each treatment group, which consisted of 11 individuals, was placed in each tank. The fish belonging to the negative control group received a basal diet (BD), whereas the fish in the treatment groups received one of the following supplements: BD+1% anthocyanins (ANTH) provided by sour cherry extract, BD+1% synbiotics (SYN) provided by fermented corn, BD+1% fermentable oligosaccharides (fOS) provided by sweet red pepper seed extract, and BD+1% carotenoids (CAR) provided by sweet red pepper pulp extract. The treatments were set up in a completely randomized design. The experiment lasted for 42 days.

The experiment was performed in a water recirculation system provided with mechanical and aerated biofilters and UV lamps. The water volume of the circular plastic tanks was 350 L.

During the experiment, the oxygen saturation level was maintained at 85±0.9% by aeration stones, and the temperature was controlled at 23.51±0.5°C. The photoperiod consisted of 12 hours of light and 12 hours of darkness. The water temperature, pH, total dissolved solids (TDS, HANNA HI98130), dissolved oxygen (DO, HACH HQ30d), and NO_2_^-^, NO_3_^-^ and NH_4_^+^ concentrations (HACH DR3900) were checked daily. Any uneaten feed and faeces were removed daily.

Prior to the experiment, the basal diet was supplemented with four different feed additives at 1%. The composition of the experimental diets is provided in S1 Table. The fish were fed manually three times each day (0800, 1200 and 1600), and the feeding rates were equal to 3% of the total biomass.

The wet body weight (BW) of the common carp individuals was measured at the beginning and end of the feeding trial (Table 1). The survival of the fish was 100% during the experiment. The growth performance and feed conversion were estimated by calculating the weight gain (WG, %), specific growth rate (SGR, %) and feed conversion ratio (FCR) of the fish using the following formulas:

- WG (%) = (Wf–Wi)/Wi x 100%, where Wf is the final wet body weight (g) and Wi is the initial wet body weight (g);
- SGR (%) = (lnWf–lnWi)/t x 100, where Wf is the final wet body weight (g) and Wi is the wet initial wet body weight (g), t is the time (day); and
- FCR (g/g) = F/(Wf-Wi), where F is the feed intake, Wf is the final wet body weight (g) and Wi is the initial wet body weight (g).

**Table 1:** Growth performance of common carp juveniles at the end of the feeding trial (means±SE, n = 3), and ABW at the beginning (_b), and end (_e) of experiment.

	Treatments				
	BD	ANTH	SYN	fOS	CAR
Survival (%)	100±0.00	100±0.00	100±0.00	100±0.00	100±0.00
ABW_b (g)	123.39±44.37	123.97±57.04	123.67±57.21	123.12±45.59	123.09±46.36
ABW_e (g)	227.67±78.51	226.22±63.20	237.11±72.74	251.67±59.86	254.89±76.59
SGR (% day^-1^)	1.46±0.20	1.43±0.27	1.55±0.09	1.70±0.15	1.73±0.10
FCR (g day^-1^)	2.21±0.42	2.26±0.55	2.04±0.12	1.80±0.22	1.76±0.14

BD: basal diet (negative control), ANTH: BD+1% anthocyanins provided by sour cherry extract, SYN: BD+1% synbiotics provided by fermented corn, fOS: BD+1% fermentable oligosaccharides provided by sweet red pepper seed extract, CAR: BD+1% carotenoids provided by sweet red pepper pulp extract. The results in the same row not sharing common superscripts are significantly different (p>0.05).

Statistical analyses of the growth performance and feed conversion were performed using the SPSS/PC+ software package. The variance homogeneity was tested by Levene’s test, and a P value higher than 0.05 was considered to indicate homogeneity. The effects of the treatments on the BW, WG, SGR and FCR results were assessed by one-way ANOVA. The significance of the differences was determined using Tukey’s multiple comparison test, and P<0.05 was considered to indicate significance.

### Determination of natural feed additives

The ANTH supplement was prepared as described by Nemes et al. [36]. Anthocyanins were extracted from Hungarian sour cherry (*Prunus cerasus*). Cherries were deseeded and homogenized, and a methanol:water:acetic acid solution at a ratio of 25:24:1 was then used to extract the anthocyanins. The sample was mixed with a magnetic stirrer (MSH 300, BioSan, Riga, Latvia) for 1 hour. After filtration, centrifugation was performed at 10,000 RPM for 5 minutes, and a simple fraction was then obtained in preconditioned tubes (Supelclean ENVI-18 SPE tubes). For preconditioning, 5 mL of MeOH and 5 mL of H_2_O were mixed with 1 mL of the fruit sample. Elution was conducted with methanol containing 20% H_2_O, and vaporization was performed at 40°C. The sample was dried in a vacuum to yield a powder. A VWR-Hitachi ChromasterUltraRs UHPLC system (Hitachi, Tokyo, Japan) with a Phenomenex Kinetex® column (2.6 μm, XB-C18, 100 A, 100 x 4.6 mm) (Phenomenex, Torrance, CA, USA) was used to determine the anthocyanin profile. Two solvents were applied for gradient elution: A, which consisted of MeOH, and B, which consisted of 3% formic acid. Elution was conducted using the following parameters: 0 minutes, 15% solvent A; 0–25 minutes, 30% solvent A; 25–30 minutes, 40% solvent A; and 30–40 minutes, 50% solvent A. UV-VIS detection was performed at 534 nm, the flow rate was maintained at 0.7 mL/minutes at 25°C, and the injection volume was 10 μL. The UHPLC profiles of the anthocyanins and the identified main compounds, including the relative areas and retention times, are shown in S1 Fig.

The synbiotics in the SYN supplement include probiotics (*Bifidobacterium bifidum*, *B*. *infantis*, *B*. *lactis*, *B*. *longum*, *Lactobacillus acidophilus*, *L*. *buchneri*, *L*. *casei*, *L*. *paracasei*, *L*. *plantarum*, *L*. *salivarius*, and *L*. *lactis*), prebiotics (fructo-, xylo-, and mannooligosaccharides and arabinogalactan), vitamins (B group vitamins and vitamins C, D2, D3, E and K2), unsaturated fatty acids (ω-3, ω-6, and ω-9), minerals/trace elements (sodium, potassium, calcium, iodine and phosphorous) and lactose. The GC profile of the oligosaccharides and the identified monomer units with the greatest relative areas and retention times are shown in S2 Fig.

The carotenoids in supplemental CAR were determined as described by Remenyik et al. [33] and Csernus et al. [37]. Carotenoids were extracted from Hungarian sweet red pepper (*Capsicum annuum*) powder (1–5 g) using a mixture of dichloroethane:acetone:methanol at a ratio of 2:2:1 as the solvent. The mixture was stirred in an ultrasonic water bath for 30 minutes and purified through Munktell-292 filter paper (VWR International, Debrecen, Hungary). For additional purification, a 0.22-μm PTFE syringe filter (TPP Techno Plastic Products AG, Switzerland) was applied. The filtered sample was vaporized at 40°C and 0.2 bar and then dissolved in an HPLC pigment reagent (isopropanol:acetonitrile:methanol at a ratio of 55:35:10) (Merck, Darmstadt, Germany). HPLC separation was conducted with a Phenomenex Kinetex® column (2.6 μm, XB-C18, 100 A, 100x4.6 mm) (Phenomenex, Torrance, CA, USA) using two elution gradients of elution: A, 11% methanol; and B, isopropanol:acetonitrile:methanol (55:35:10 V/V/V%) mixture. The gradient elutions were performed with the following settings: 0–3 minutes, 100% solvent A; 15–20 minutes, 20% solvent A; 25–45 minutes, 100% solvent B; and 48–50 minutes, 100% solvent A. For sample detection, a diode array detector (DAD) was used with a flow rate setting of 0.6 mL/minutes. The sample was injected in a 10-μL volume, and DAD detection was applied at 460 and 350 nm. The HPLC profile of the carotenoids and identified monomer units with the greatest relative areas and retention times are shown in S3 Fig.

The fermentable oligosaccharides in the fOS supplements was determined as described by Csernus et al. [37] Hungarian sweet red pepper seeds (*Capsicum annuum*) were used for the extraction of fermentable oligosaccharides with a high arabino-galactose content. An HP 5890 gas chromatograph with an SP-2380 capillary column (30 m x 0.25 mm, 0.2 μm) was used to measure the composition of oligosaccharides. The samples were lyophilized and extracted with trifluoracetic acid:acetic acid:water at a ratio of 5:75:20 as the solvent. A reduction step was used to convert the oligosaccharides into alditol-acetate with NaBH_4_ at alkaline pH. The sugars were then converted to sugar alcohols (alditols) with acetic anhydride in pyridine, which removed interfering isomers and anomers. The nitrogen gas flow rate was 1.2 mL/min. The injector temperature was set to 300°C with a split ratio of 1:20. A flame ionization detector (FID) was applied for the identification of oligosaccharides. The GC profiles of the oligosaccharides and the identified monomer units, including the greatest relative areas and retention times, are shown in S4 Fig.

### Sample collection

During the experimental period, intestinal samples were obtained from the fish at ages of 31 weeks (day 0), 33 weeks (14^th^ day), 35 weeks (28^th^ day), and 42 weeks (42^nd^ day). The average body weight (ABW) was measured at the beginning and end of the feeding period (Table 1). The fish were euthanized with clove oil [38] and disinfected with 96% EtOH, and the whole intestine was placed into a tube. During the gut collection procedure, sampling was also performed from the air and surgical table to monitor the environmental contamination, which can bias sequencing. The bowel was stored and transported on ice in sterile PBS (Thermo Fisher Scientific, MD, USA). Residual tissue was removed from the guts under class II laminar flow hood (Thermo Fisher Scientific, MD, USA), and a 10-g sample was digested in 100 mL of lysis buffer (500 mM NaCl, 50 mM Tris HCl, 50 mM EDTA, 4% SDS, 0.1 mg of Proteinase-K, and 10% Triton X) at 56°C for 16 hours.

### Sample preparation and mechanical cell lyses

The lysate was pipetted into a 50-mL centrifuge tube. The samples were centrifuged at 2,000 x g for 5 minutes, and the upper colloidal phase was discarded. The middle phase was transferred to a new 50-mL centrifuge tube, and PBS was added to the lysate at a ratio of 1:1. The samples were shaken for approximately 3 minutes, the mixture was centrifuged at 500 x g for 5 minutes, and the supernatant was collected in a sterile 50-mL centrifuge tube. This step was repeated three times. We performed mechanical cell lysis: 1,000 μL of sample was added to a PowerBead tube (Qiagen, Hilden, Germany) and lysed using a MagNA Lyser (Roche Applied Sciences; Penzberg, Germany) at 5,000 RPM for 30 seconds. The samples were centrifuged at 16,000 x g for 1 minute, and the supernatant was pipetted into a sterile Eppendorf tube.

### DNA extraction

The conventional isolation method was used for total bacterial genomic DNA extraction. Eight hundred microliters of phenol:chloroform:isoamyl alcohol (25:24:1) (Thermo Fisher Scientific, MD, USA) was mixed with 800 μL of lysate, and the mixture vortexed thoroughly for approximately 15 seconds. After sample homogenization, we incubated the samples at room temperature for 3 minutes and centrifuged them for 10 minutes at 16,000 x g and 4°C. The upper aqueous layer was carefully collected into a new sterile Eppendorf tube. For DNA precipitation, a mixture of 1 μL of glycogen (20 μg/μL), ammonium acetate (0.5x volume, 7.5 M NH_4_OAc) and 100% ethanol (2.5x volume) was added to the supernatant. Samples were incubated at -70°C for 16 hours and then centrifuged them for 30 minutes at 16,000 x g and 4°C to pellet the DNA. We carefully discarded the supernatant without disturbing the pellet, and 500 μL of 70% EtOH was added to the sample. The mixture was shaken for 20 seconds and centrifuged at 4°C and 16,000 x g for 5 minutes, and the supernatant was carefully removed. This washing step was repeated twice. We dried the DNA pellet at room temperature and then resuspended it in 15 μL of nuclease-free water. The DNA concentrations were determined using a Qubit® Fluorometric Quantitation dsDNA assay kit (Thermo Fisher Scientific, Waltham, MA, USA) and a Clariostar microplate reader (BMG Labtech, Ortenberg, Germany). The DNA quality and quantity were confirmed using a Nanodrop 2000 Spectrophotometer (Thermo Fisher Scientific), and the DNA integrity (shearing/fragmentation) was measured with a 4200 TapeStation System (G2991AA, Agilent Technologies; Santa Clara, CA, USA). The DNA elutes were stored at –20°C.

### Library construction and sequencing

Library preparation was performed according to the standard Illumina (San Diego, CA, USA) 16S metagenomic sequencing protocol (15044223 Rev. B). The V3 and V4 hypervariable regions of the bacterial 16S rRNA gene were targeted to generate amplicons of ~460 using the universal primer set 341F (5’ CCTACGGGNGGCWGCAG 3’) and 785R (5’ GACTACHVGGGTATCTAATCC 3’) flanked by Illumina overhang adapter sequences (forward overhang: 5’ TCGTCGGCAGCGTCAGATGTGTATAAGAGACAG 3’, reverse overhang: 5’ GTCTCGTGGGCTCGGAGATGTGTATAAGAGACAG 3’) (Sigma Aldrich, MO, USA). After completion of the amplicon PCR with 2x KAPA HiFi HotStart ReadyMix, dual indexing of the samples with adaptor sequences (i7-N7xx-12 items, i5-S5xx-8 items) was performed using the Illumina Nextera XT Index Kit (FC-131-1001/2). PCR clean-up and amplicon size selection were performed using with KAPA Pure Beads (KAPA Biosystems) based on the technical data sheet (KR1245 –v3.16) provided by the manufacturer, which resulted in final ~580-630-bp libraries. For each case, verifications were performed with PCR Agilent D1000 screen tapes (5067–5582) and D1000 reagents (5067–5583). The 16S amplicon libraries for each sample were quantified by qPCR, normalized with respect to the amplicon sizes and pooled into a single library at equal molar quantities. Finally, 5 μL of the 4 nM DNA library pool was prepared for sequencing on the Illumina MiSeq platform. The library pool was denatured with 0.2 M NaOH and diluted to a final concentration of 6 pM. Sequencing was performed with a MiSeq Reagent Kit v3–618 cycle (MS-102-3003) following the manufacturer’s protocols (Illumina, Inc., San Diego, CA, USA). Paired-end sequencing (2x301 nt) was performed on an Illumina MiSeq platform with 5% PhiX spike-in quality control (PhiX Control Kit v3—FC-110-3001).

### Preparing sequence reads for downstream analysis

To demultiplex the paired end reads and construct FASTQ files, Illumina BaseSpace software was used. The data were analysed using the Quantitative Insight Into Microbial Ecology pipeline (QIIME2, ver 2019.1) [39]. Cutadapt software integrated in QIIME2 software was used to check for the presence of adapter sequences (CTGTCTCTTATACACATCT) and trim the 3’ end of the reads. Quality trimming, filtering and chimera removal were performed with DADA2 software [40]. The trimming parameters were set as follows: the forward read length was set to 299 bases; for the reverse reads, the length was set to 249 bases. No bases were cropped from the 3’ end of the forward and reverse reads.

### Bioinformatic analyses

QIIME2 integrated in MAFFT software was used for multiple sequence alignment [41], and the reads were taxonomically classified using a Naïve Bayesian classifier trained on the Greengenes (ver13_8) [42] reference database by selecting mapping points according to the forward-reverse primer set that was used for amplifying the V3-V4 regions of the 16S rRNA genes of the bacterial community (341F, 806R). Phylogenetic trees were constructed with the FastTree plugin [43]. The QIIME2 pipeline was used for the alpha diversity analyses. For sample normalization, a read depth of 1494 was set. To analyse the alpha diversity, Shannon’s index [44, 45], Faith’s phylogenetic diversity index [46], Simpson evenness [47], and Chao-1 index [48] were calculated using the QIIME2 pipeline. The differences in the alpha diversity were assessed using the Kruskal-Wallis test. To estimate beta diversity differences weighted UniFrac distance [49] was calculated. For visualization of beta diversity distance-based dissimilarity matrices PCoA plots were generated using the Emperor plugin [50]. Beta diversity group significances were calculated with Permutational multivariate analysis of variance (PERMANOVA) pseudo-F statistical test [51]. QIIME2 artifact files were exported from the pipeline and converted to TSV files that could be used with different visualization packages. We used the Python (ver 3.6.5) Seaborn package to generate heatmaps, donut plots, and boxplots were constructed with the pandas and matplotlib packages. Correlational analyses were performed with the Corrplot R package.

## Results

### Growth performance

To investigate the effects of phytonutrients on the growth performance of *Cyprinus carpio*, the average body weight (ABW) was measured (Table 1). At the beginning of the feeding experiment, the ABW was 123.45±49.28 g, whereas at the end of the experiment, the ABW reached 239.51±70.25 g. We estimated that the phytonutrients -based diet had no significant effect (p>0.05) on the ABW of common carp juveniles. The highest ABW was observed in the CAR animals (254.89±76.95 vs. 227.67±78.51 g for the controls). The supplementation of the diet with plant extracts did not have an impact on the specific growth rate (SGR) or feed conversation ratio (FCR) of the fish. However, the highest SGR (1.73±0.10% day^-1^) values and the best FCR results (1.76±0.14 g^-1^) were observed with the CAR treatment (Table 1).

### Correlation between average body weight and fish microbiota

**Fig 2:**
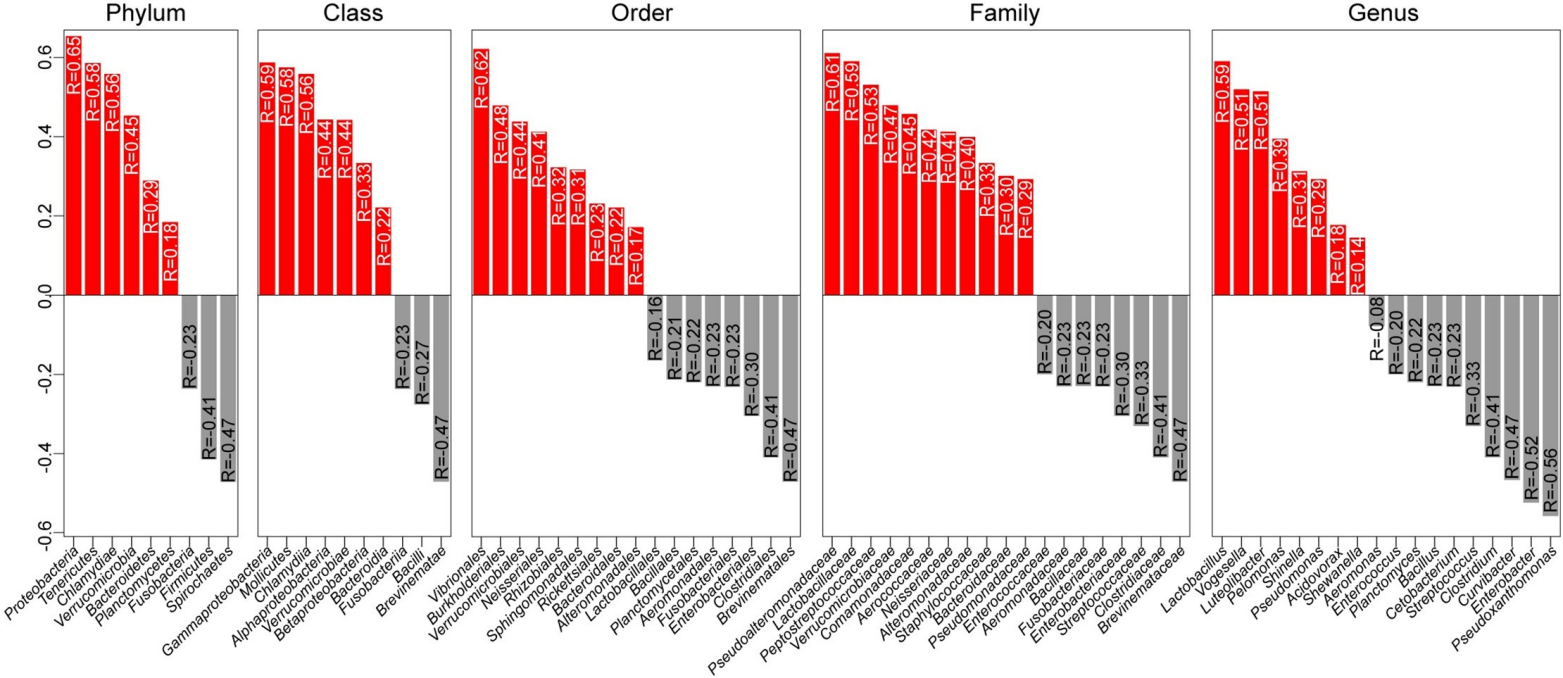
Spearman correlation analysis was performed to measure the associations between the ABW and the intestinal taxa of *Cyprinus carpio*. The correlation values range from –1 to +1 and indicate the level of consistency of the positive (≥0; red) and negative (<0; grey) correlations. The correlations were calculated at five taxonomic levels: phylum, class, order, family, and genus.

We managed to identify taxa showing correlations with body weight. Specifically, we captured notable associations between the GIT microbiota and the body weight of *Cyprinus carpio*. Alterations in the strength and direction of the correlations were measured at five taxonomic levels (Fig 2). At the phylum level, *Proteobacteria* (R = 0.65), *Tenericutes* (R = 0.58) and *Chlamydiae* (R = 0.56) showed the strongest positive correlations, and the phyla *Spirochaetes* (R = -0.47) and *Firmicutes* (R = -0.41) showed the most notable negative correlations with body weight gain. Further important positive correlations were observed for the classes *Gammaproteobacteria* (R = 0.59), the order *Vibrionales* (R = 0.62), the families *Pseudoalteromonadaceae* (R = 0.61) and *Peptostreptococcaceae* (R = 0.53) and the genera *Lactobacillus* (R = 0.59) and *Luteolibacter* (R = 0.51). In contrast, striking negative correlations were detected between the ABW and the class *Brevinematae* (R = -0.47), its order *Brevinematales* (R = -0.47) and its family *Brevinematacea* (R = -0.47) and with the order *Clostridiales* (R = -0.41), the family *Clostridiaceae* (R = -0.41) and the genera *Enterobacter* (R = -0.52) and *Pseudoxanthomonas* (R = -0.56).

### Ageing- and diet-driven alterations in the alpha and beta diversities

**Fig 3:**
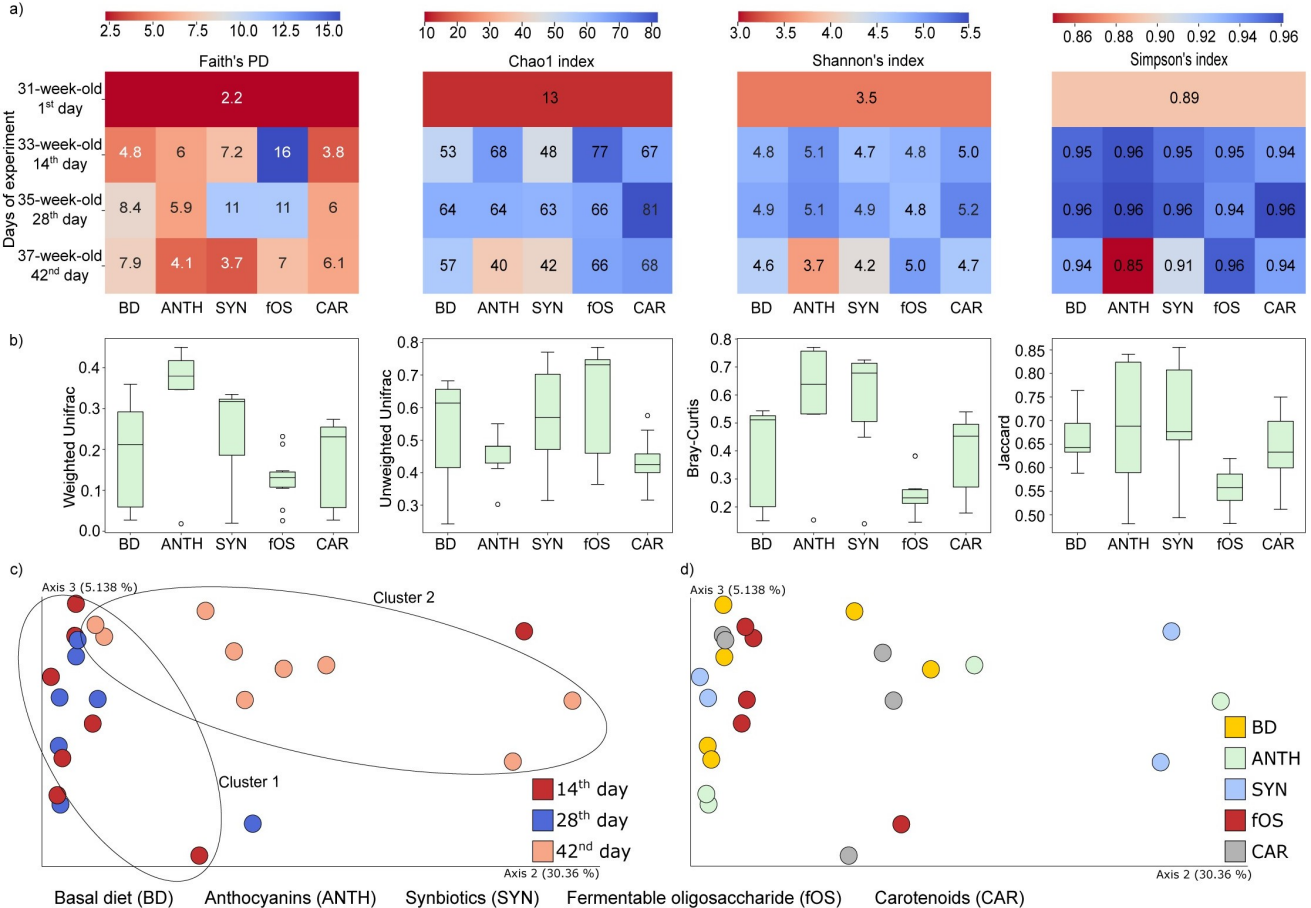
Alpha diversity metrices used in the comparison analysis. a) Annotated heatmaps showing the values of Faith’s PD, Chao-1, Shannon’s and Simpson’s diversity indexes as a function of the time of sample collection (y axis) and the experimental settings (x axis). b) The sample distances were calculated based on quantitative (Bray-Curtis, weighted UniFrac) and qualitative (Jacquard, unweighted UniFrac) dissimilarity-based statistics c, d) Weighted UniFrac analysis was performed to identify ageing- and treatment-driven differences between the groups. The beta diversity relationships are summarized in two-dimensional scatter plots. The distances between dots are representative of differences in microbiota compositions. BD represents fish that received a basal diet, which served as a negative control with no dietary supplement or the following dietary treatments as supplemented feed: BD+1% anthocyanins (ANTH) provided by Hungarian sour cherry extract, BD+1% synbiotics (SYN) provided by fermented corn, BD+1% fermentable oligosaccharides (fOS) provided by Hungarian sweet red pepper seed extract, and BD+1% carotenoids (CAR) provided by Hungarian sweet red pepper pulp extract.

Microbial diversity is considered a key factor influencing animal productivity through disease tolerance. Diverse microbiota shows increased resilience, and only tendentious shifts can push them toward an unhealthy state. Alpha and beta diversity metrics were calculated to track the notable conversions in the community diversity of the control (BD) and treatment groups (ANTH, SYN, fOS, and CAR) (Fig 3). Specifically, Faith’s PD, Chao-1, Shannon and Simpson diversity indices were used to evaluate the species abundance, richness and evenness of the GIT microbiota. Annotated heatmaps represent the community alpha diversities in relation to the time of sampling and treatments (Fig 3A). In the case of ANTH, SYN, and fOS treated samples the analysis of Faith’s PD showed a steady increase by week 4 (day 28^th^) of the study period followed by a sharp decline at week 6 (day 42^nd^) of the experimental period (mean Faith’s PD: 9.3 > 4.9). Chao-1, Shannon’s, and Simpson’s indices did not show a marked decrease after the 4th week (day 28^th^). Interestingly, the most pronounced decline in the community alpha diversity was observed with the ANTH supplementation during the last 2 weeks of the study period. Moreover, the lowest value of Simpson’s index (day 42^nd^: 0.85 vs. day 1^st^: 0.89) was detected during the last quarter of the experimental period. In general, sweet red pepper seed extract (fOS) was able to ameliorate the community diversity according to Faith’s PD and Chao-1 indices, and this treatment group was the only group that did not show a decline in Shannon’s and Simpson’s indices during the last 2 weeks of the study period.

Four beta diversity metrics were investigated; specifically, the weighted and unweighted UniFrac, Bray-Curtis, and Jaccard distances, (Fig 3B) between the different experimental groups were determined. Distance-based dissimilarity matrices showed that nutraceuticals did not exert a significant (p<0.05) influence on the overall community variations between the different treatment groups.

Principal coordinate analysis (PCoA) using weighted UniFrac distances was performed to measure the age dependency of the community taxonomy data, and this analysis yielded two clusters (clusters 1 and 2) representing different spatial ordinations between the fishes at days 14^th^ (aged 33 weeks), 28^th^ (aged 35 weeks) and 42^nd^ (aged 37 weeks) (Fig 3C). The microbiota of 37-week-old *Cyprinus carpio* showed a distinct clustering pattern compared with the microbiota at earlier time points (aged 33–35 weeks) (Fig 3C). When marking the samples according to diet, no distinct patterns were observed between the treatment groups (Fig 3D). Based on the PCoA plots, we concluded that age induced more pronounced shifts in the microbial community than diet.

### Characterization of the *Cyprinus carpio* gut symbiome

**Fig 4:**
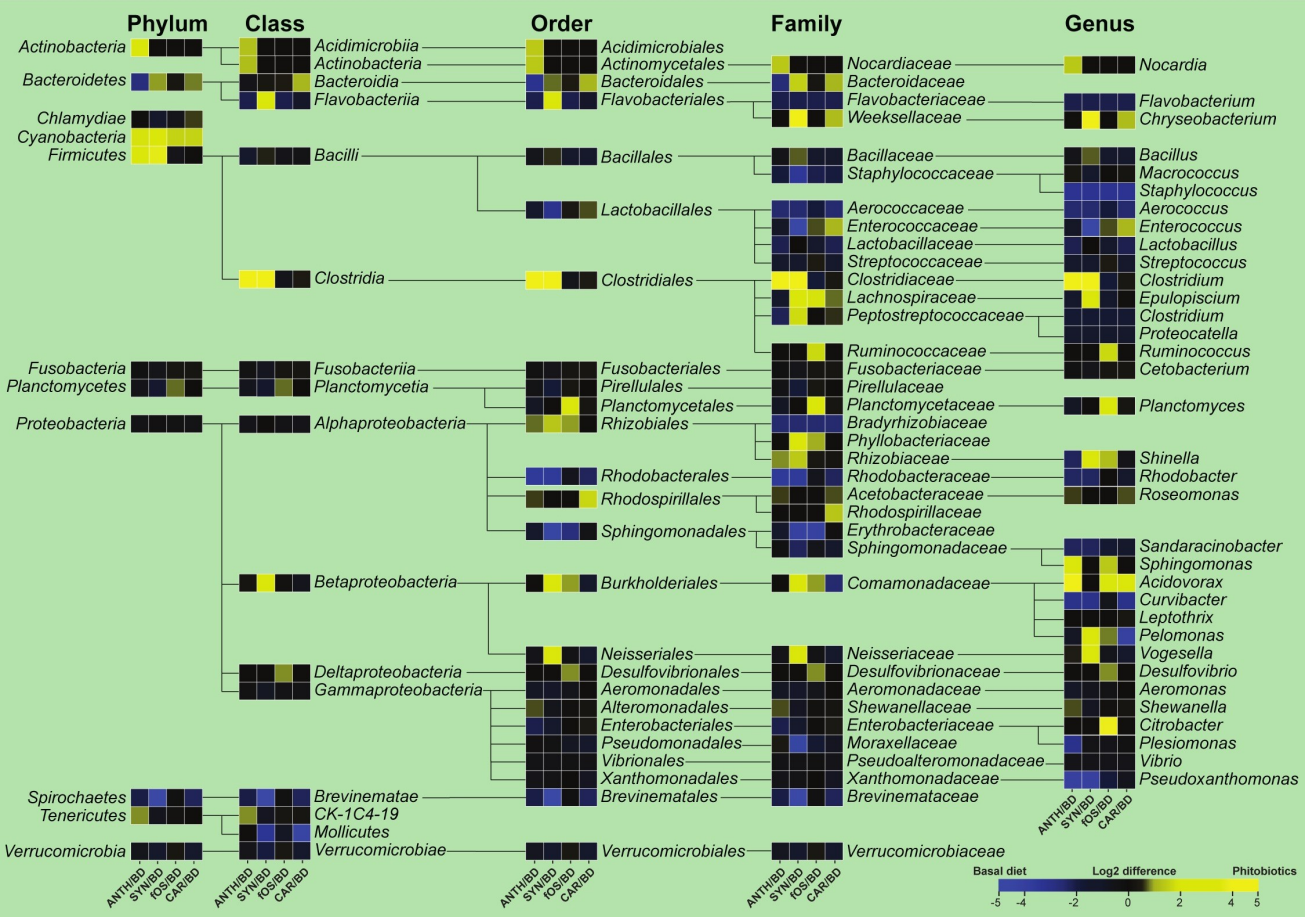
The composition of the healthy *Cyprinus carpio* GIT symbiome was mapped. Abundance variations were observed in key taxa. A composite heat map was created to note the pronounced distortions in the taxonomic profiles based on the normalized abundance data (ANTH/SYN/fOS/CAR vs. BD). BD represents fish that received a basal diet, which served as a negative control with no dietary supplement or the following dietary treatments as supplemented phytonutrients feed: BD+1% anthocyanins (ANTH) provided by Hungarian sour cherry extract, BD+1% synbiotics (SYN) provided by fermented corn, BD+1% fermentable oligosaccharides (fOS) provided by Hungarian sweet red pepper seed extract, and BD+1% carotenoids (CAR) provided by Hungarian sweet red pepper pulp extract. The extents of the differences are illustrated on the composite heatmap with gradient colours, where the yellow scale shows the taxa found at higher abundances in the phytonutrients-treated specimens (log2(phytonutrients/BD)>0) and the blue scale indicates a higher taxa abundance in the non-treated controls (log2 (phytonutrients/BD) < 0). Shifts in the abundances of 122 taxa due to phytonutrients can be observed.

Our interest in understanding phytonutrient-induced taxonomic shifts in the symbiotic microbiota of cultured, healthy *Cyprinus carpio* has grown. The observed community compositions relative to that of the healthy controls are shown in Fig 4. Differences in normalized abundance data were attained by considering 11 phyla, 16 classes, 25 orders, 36 families and 34 genera. Graphical representations were generated by calculating the log2 differences in taxa abundances between fish fed phytonutrient-supplemented diets (ANTH, SYN, fOS, and CAR) and those given the basal diet (BD). Taxonomic shifts were obtained when comparing untreated vs. treated healthy *Cyprinus carpio* symbiomes.

Nutraceuticals favoured the growth of *Cyanobacteria*, and anthocyanins (ANTH) induced the growth of the phylum *Actinobacteria*, the family *Nocardiaceae*, and the genera *Sphingomonas* and *Acidovorax*. In contrast, synbiotics (SYN) stimulated the classes *Flavobacteria* and *Betaproteobacteria*, the orders *Burkholderiales* and *Neisseriales*, the families *Weeksellacea*, *Peptostreptococcaceae*, *Phyllobacteriaceae* and *Neisseriaceae* and the genera *Chryseobacterium*, *Epulopiscium*, *Pelomonas*, and *Vogesella*. Anthocyanins (ANTH) and synbiotics (SYN) induced marked enrichment of *Firmicutes*, *Clostridia*, *Clostridiales*, *Clostridiaceae* and *Clostridium*. The abundances of the families *Lachnospiraceae* and *Phyllobacteriaceae* and the genus *Shinella* were increased by synbiotics (SYN) and fermentable oligosaccharides (fOS). Fermentable oligosaccharides (fOS) induced the famillies *Ruminococcaceae* and *Plantomycetaceae*, and the pulp extract (CAR) favoured the growth of the class *Bacteroidia* and order *Bacteroidales* and the families *Bacteroidaceae*, *Rhodospirillaceae*, and the genera *Chryseobacterium*, *Enterococcus*, and *Acidovorax*.

### Diet affects the distribution of beneficial and potentially pathogenic and spoilage bacteria

The beneficial or detrimental effects of microorganisms on the intestinal microbiota can be strongly influenced by several factors, such as the fish species, prebiotic substrates, composition of the complex microbial populations, pH, living environments, and seasons. Because both biotic and abiotic stressors decrease the abundances of beneficial bacteria, opportunistic members of the community might take advantage and become infectious [52], leading to serious seasonal losses in aquaculture.

**Fig 5:**
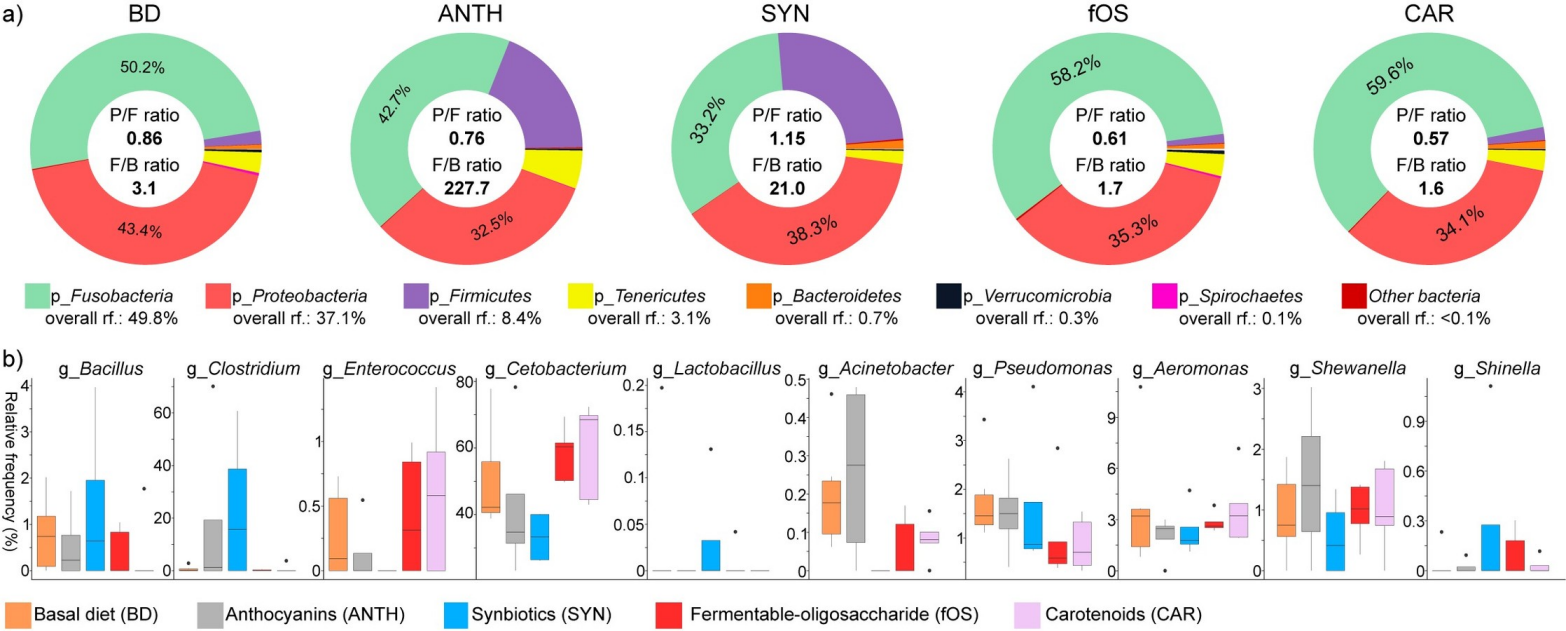
Diets enriched in phytonutrients affect important beneficial and potentially detrimental members of the *Cyprinus carpio* GIT. a) Donut plots represent diet-specific distortions in the main phyla. The *Firmicutes*-to-*Bacteroidetes* ratio (F/B ratio) and *Proteobacteria*-to-*Firmicutes* ratio (P/F ratio) are shown. b) Boxplots allow comparisons of the relative proportions of important general; specifically, relevant genera in the different groups are represented as boxplots. BD represents fish that received a basal diet, which served as a negative control with no dietary supplement or the following dietary treatments as supplemented feed: BD+1% anthocyanins (ANTH) provided by Hungarian sour cherry extract, BD+1% synbiotics (SYN) provided by fermented corn, BD+1% fermentable oligosaccharides (fOS) provided by Hungarian sweet red pepper seed extract, and BD+1% carotenoids (CAR) provided by Hungarian sweet red pepper pulp extract.

High-throughput sequencing of the bacterial 16S rRNA genes indicated that a phytonutrient-enriched diet induced significant shifts in the core phyla of the *Cyprinus carpio* GIT (Fig 5). During this feeding experiment, the carp gut was dominated by *Fusobacteria* (Σ49.8±16.6%) and *Proteobacteria* (Σ37.1±16.0%), and significant differences were found between the treatment groups (Fig 5A). The relative frequencies of the two dominant phyla showed the greatest most equalization in the fermented corn-treated (SYN) samples and were most pronounced in the pepper seed (fOS, P/F ratio: 0.61, 35.3% *Proteobacteria*, 58.2% *Fusobacteria*)- and pepper pulp extract-treated samples, (CAR P/F ratio: 0.57, 34.1% *Proteobacteria*, 59.6% *Fusobacteria*).

By modulating the optimal nutrition and energy utilization of the host, the phyla *Bacteroides* and *Firmicutes*, which are associated with SCFA synthesis, can improve the growth performance of aquatic animals [53]. According to data, elevated *Firmicutes* levels can be associated with increased nutrient absorption, whereas *Bacteroidetes* is correlated with enhanced hydrolysis of glycogen, starch and polysaccharides. Under our experimental settings, the highest and lowest F/B ratios were obtained with the ANTH-fed fish (227.7) and the CAR-fed carp (1.6), whereas the control animals (BD) had an F/B ratio of 3.1.

Anthocyanins increased the abundance of *Shewanella* (ANTH 1.4±1.3% vs. BD 0.9±0.7%) in comparison to that obtained with the basal diet. Synbiotics increased the abundances of *Bacillus* (SYN 1.3±1.8% vs. BD 0.8±0.8%) and *Shinella* (SYN 0.2±0.5% vs. BD 0.04±0.09%) and decreased that of *Shewanella* (SYN 0.5±0.6% vs. BD 0.9±0.7%). The genus *Acinetobacter*, which contains potential fish pathogen species, was not detected in the synbiotic-fed (SYN) fish, whereas the pepper pulp (CAR 0.1±0.03%) and seed extracts (fOS 0.06±0.08%) decreased the abundance of this genus, and the sour cherry extract (ANTH 0.1±0.2%) significantly increased its abundance relative to that found in fish fed the basal diet (BD 0.3±0.1%).

According to other data, *Cetobacterium* is involved in the fermentation of peptides and carbohydrates [54], including in the production of vitamin B12 [15]. Pulp extracts increased the abundances of *Cetobacterium* (CAR 59.6±14.5% vs. BD 50.2±15.6%) and *Enterococcus* (CAR 0.6±0.6% vs. BD 0.3±0.3%) and decreased the abundances of *Bacillus* (CAR 0.3±0.7% vs. BD 0.8±0.8%), *Pseudomonas* (CAR 0.9±0.5% vs. BD 1.8±0.8%), and *Acinetobacter* (CAR 0.1±0.04% vs. BD 0.3±0.2%). The genus *Shinella*, which can potentially eliminate nitrate contamination from the environment, was markedly increased in the synbiotic-fed fish.

### Comparative metagenomics reveals taxonomy associations in carp symbiomes

**Fig 6:**
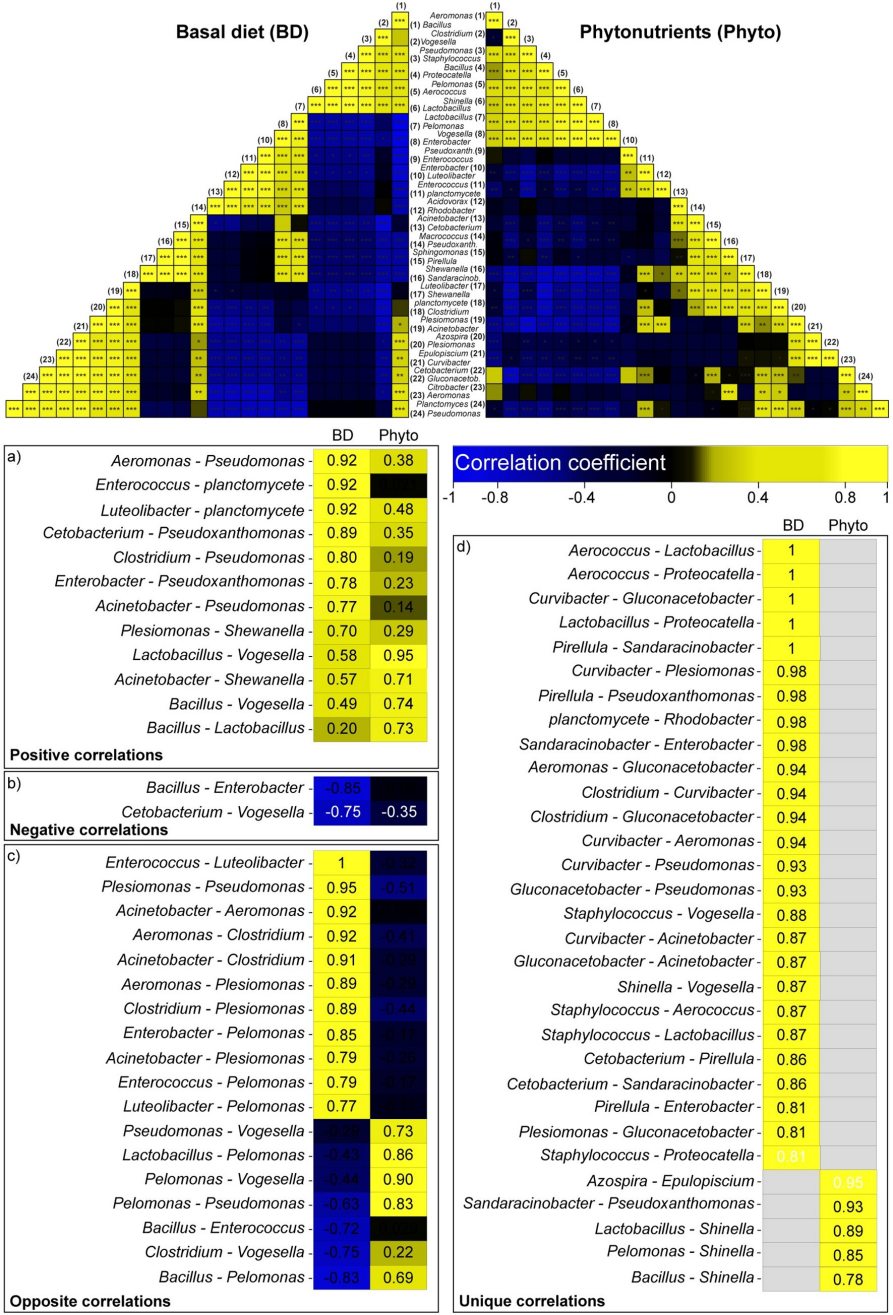
Convergent and divergent abundance patterns identified in *Cyprinus Carpio* symbiomes. Spearman’s correlations were calculated between the 24 most abundant genera in the basal diet-fed control and phytonutrients-fed carp. For better visualization, we listed associations with similar (“positive correlations” and “negative correlations”) and different (“opposite correlations”) correlation precursors in the two experimental categories. Unique correlations are also shown. BD represents fish that received a basal diet, which served as a negative control with no dietary supplement. The following dietary treatments were supplemented as phytonutrients: BD+1% anthocyanins (ANTH) provided by Hungarian sour cherry extract, BD+1% synbiotics (SYN) provided by fermented corn, BD+1% fermentable oligosaccharides (fOS) provided by Hungarian sweet red pepper seed extract, and BD+1% carotenoids (CAR) provided by Hungarian sweet red pepper pulp extract.

Taxonomic interconnections were revealed between and within the GIT microbiota of the control and phytonutrient-fed fish. We also estimated the extent to which genera are likely to play an indispensable role in maintaining the gastrointestinal health of *Cyprinus carpio*. Relative proportions were correlated using Spearman’s correlation method (Fig 6). The analysis revealed 32 significant correlations between genera, and these included 12 positive (Fig 6A), two negative (Fig 6B), and 18 opposite (Fig 6C) correlations. Furthermore, 31 unique (26 in BD, five in phytonutrients-fed fishes) significant correlations were found between *Cyprinus carpio* GIT genera (Fig 6D). Significant positive associations were found among *Planctomycetes*, *Enterococcus*, and *Luteolibacter*, among *Pseudoxanthomonas*, *Enterobacter*, and *Cetobacterium*, and among *Pseudomonas*, *Acinetobacter* and *Aeromonas*. However, *Enterobacter* and *Cetobacterium* were negatively correlated with *Bacillus* and *Vogesella*, respectively.

Notably, opposite correlations were detected between *Enterococcus* and *Luteolibacter*, between *Pseudomonas* and *Plesiomonas* and among *Clostridium*, *Aeromonas*, *Acinetobacter* and *Plesiomonas*.

We also revealed significant correlations between genera that were unique to a specific diet. The basal diet-fed animals showed the highest number of unique taxonomy matches (26 positive correlations). As such, very strong associations between *Lactobacillus* and *Aerococcus* and among *Proteocatella*, *Curvibacter* and *Gluconacetobacter* were exclusively obtained in nontreated carp.

## Discussion

Aquaculture fish production has replaced the use of capture fisheries since the global demands for fish have surpassed those for beef, pork and poultry products [2, 55]. At present, one-fifth of the world’s population has opposed the use of fisheries, but fish stocks are becoming increasingly overexploited [56]. The establishment of a more sustainable aquaculture sector is needed to supply this high-quality protein to rapidly growing world’s population [57].

Controlling the outbreaks of infections associated with high stocking densities of fish is essential. The routine usage of antibiotics in aquacultures releases significant amounts of drug residues and potentially increases resistance in natural environments. Antibiotic-free breeding systems have become more important in animal farming and in easing pollution and resistance spread. [58–60].

Most of the microbes that can cause infections in aquacultures are part of the healthy baseline fish microbiota. Therefore, their complete elimination from fish rearing systems is not achievable. Additionally, by carrying resistance against a wide range of antibiotics, their zoonotic importance is about to escalate [61]. Although the mechanisms underlying “colonization resistance” are unclear, it has been suggested that microbiota compete with pathogens for niches by producing and secreting antimicrobial peptides [14].

As a substitute for antibiotics, the use of natural alternatives for farmed fish via dietary supplementation is a key area of research [62, 63]. The results of other studies have shown that probiotic-based supplements might facilitate this process [5]. However, only a few recent studies on the application of phytonutrients as feed additives in aquaculture settings have provided a detailed analysis. The available data propagate the importance of the dietary application of plant-derived bioactive components as potential growth promoters that support intestinal homeostasis by modulating feed utilization. However, further investigations are needed to understand how these natural compounds are beneficial to aquatic animals.

Common carp is one of the most commonly cultivated species in fish rearing systems worldwide [64]. Due to sustainability concerns, fish meat production continues to increase, which encourages aquaculturists to apply the latest technologies with the aim of improving the maximum yield [16].

The intestine of aquatic animals is an ideal environment for the colonization and proliferation of commensal microbes [65]. The GIT microbiome composition shapes host physiology and growth, but these functions in aquatic animal hosts have not yet been fully investigated. Only a limited number of studies have evaluated the association between the fish microbiota and production outcomes in aquaculture settings [66, 67].

This feeding experiment was conducted to decipher the phytonutrient-induced changes in the GIT microbiota of 7-month-old *Cyprinus carpio*. Next-generation sequencing targeting the 16S rRNA gene was applied to trace even non-cultivable and low-abundance rare taxa.

Our results showed that dietary supplementation did not exert a significant effect on the ABW of carp juveniles, which is in accordance with the results described by Hoseinifar et al. [68].

A high intestinal microbiota diversity is generally considered beneficial for health [69], and we thus investigated the effects of phytonutrients on the diversity of the healthy *Cyprinus carpio* symbiome. In this study, the acclimation of the *Cyprinus carpio* intestinal microbiota did not result in noticeable changes in the alpha diversity values, which implies that the healthy *Cyprinus carpio* microbiota can dynamically change the community heterogeneity to preserve basic biological functions. We noted that acclimation to the new environmental conditions involved a noticeable adjustment in the alpha diversities of the healthy *Cyprinus carpio* GIT microbial communities. At the beginning of the experiment, the average alpha diversity values were lower, but we observed a general improvement throughout the experimental period. During the study, the highest diversity was observed at the 4^th^ week of the feeding period (day 28^th^) in the animals fed sweet red pepper pulp (CAR) and sour cherry (ANTH) extracts. The Chao1 index was increased by the bioactive components in sweet red pepper seeds (fOS) and pepper pulp (CAR). Shannon’s and Simpson’s indices did not show remarkable differences over the course of the experiment.

The gut microbiota provides the host with important short-chain fatty acids (SCFAs). In fish, carbohydrate fermentation occurs mostly by members of the genus *Bacteroides*, which are known producers of SCFAs that play an important role against gut inflammation [15]. Based on our data, the highest abundances of *Bacteroidales* were found in the fermented corn (SYN)- and pepper pulp-treated (CAR) groups.

In general, due to shorter retention times, herbivorous and omnivorous carp show lower SCFA levels; therefore, pre- and probiotic supplementation might be appropriate [70]. Different fibre ratios might result in different gut microbiota structures. A proportionality is generally observed between the diversity of the intestinal microbiota and SCFA producers [71]. Additionally, stress-stimulated microbiota dysbiosis is considered a relevant factor that negatively affects the proportions of SCFA-producing microorganisms.

Specifically, synbiotics selectively promoted the growth of *Bacillus* and *Lactobacillus*. In contrast, anthocyanins and synbiotics decreased the abundances of *Cetobacterium*, whereas fermentable oligosaccharides enriched this genus.

*Clostridia* is a member of the endogenous flora of the fish intestine and is involved in pathogen exclusion through its antibacterial activity [54]. *Clostridiales* species are also associated with carbohydrate degradation and are responsible for producing SCFAs in vertebrates [72]. In this study, higher abundances of the genus *Clostridium* were found in fermented corn-fed fish compared with the controls [54].

*Enterococcaceae* are lactic acid bacteria (LAB) comprising both pathogenic and commensal microorganisms that are ubiquitous even as gut symbionts. Their competitiveness is also due to their ability to produce bacteriocins recognized for their wide-range effectiveness against pathogenic and spoilage bacteria [73]. Sweet red pepper seed (fOS) and pulp (CAR) extracts also enriched the genus *Enterococcus*, whereas sour cherry (ANTH), fermented corn (SYN) and sweet red pepper seed (fOS) extracts did not exert remarkable influence on the abundance of LAB.

We also investigated the effects of natural feed additives on the abundances of potentially pathogenic and spoilage bacteria. Intrinsic characteristics, such as high pH values and high levels of proteins and free amino acids, make fish products highly susceptible to spoilage [74]. Based on their metabolic activities in the *Enterobacteriaceae* family and ubiquitous *Shewanella*, *Pseudomonas* species can have negative effects on fish meat quality and are often responsible for the psychotropic spoilage of fish products through the production of hydrolytic enzymes [29]. In this experiment, the presence of *Pseudomonas*, which can improve the survival of pathogenic bacteria [75], in fish meat was decreased by the fructo-oligosaccharides found in the pepper extracts (fOS, CAR).

The probiotic *Shewanella*, which is one of the major omega-3-polyunsaturated fatty acid-producing genera, is often used in aquacultures. This genus can also be commonly isolated from the GIT of fish and vertebrates [76]. Nevertheless, the genera *Shewanella* and *Pseudomonas* are important psychotropic spoilage bacteria that can produce hydrolytic extracellular enzymes and might exert negative effects on fresh meat quality [74]. The highest abundance of *Shewanella* was detected in the anthocyanin extract-treated samples [14].

The known pathogen *Aeromonas* is widely distributed in freshwater aquatic environments and is also a known member of the endogenous flora of freshwater fish that participates in the fermentation of organic compounds, cellulose degradation, and antibacterial activity [54]. Intensive practices in aquacultures typically produce weakened populations of to their virulence factors, *Aeromonas* are often associated with human infections, which leads to significant seasonal financial losses [77]. *Aeromonas* are often the causative agent of motile *Aeromonas septicemia* [30]. Due to their virulence factors, *Aeromonas* are often associated with human infections [78]. *Aeromonas* have also been identified as important spoilage bacteria that deteriorate the quality of aquatic products [79]. Based on our data, the pepper pulp extracts (CAR) positively stimulated the growth of *Aeromonas*, whereas anthocyanins (ANTH) and synbiotics (SYN) decreased the abundance of *Aeromonas*. *Aeromonas* was also found to enhance growth in zebrafish [80]; however, our results showed a negative association between the relative frequency of the genus and body weight gain.

There is an indispensable need to investigate the effects of natural and bioactive components in aquacultures. Any successful implementation of an alternative, antibiotic-free meat production systems in animal farming could be beneficial and might be accompanied by high economic significance. Based on their natural sources, phytonutrients carry many advantages over traditional medicines. We demonstrated that phytonutrients positively affect the beneficious bacteria of the carp intestinal microbiota, and thus, their medical and health benefits might be important in preventing or treating infections and diseases in antibiotic-free farming systems. In summary, we believe that phytonutrients rich in minerals, vitamins, and amino acids can provide an efficient strategy for aquaculture that improves the safety of the hosts and the sustainability of fish production.

## Conclusions

Increasing studies are investigating how natural feed additives can induce positive changes in the fish GIT microbiota and support pathogen exclusion. It has been envisioned that providing information on the applications of phytonutrients in aquaculture will facilitate the development of future strategies of farmed aquatic animals.

- In this feeding experiment, we applied a culture-independent molecular approach to thoroughly discover the phytonutrient-induced alterations in the GIT microbiome of common carp. The applied phytonutrients were derived from food waste materials, which emphasizes the economic potential of this strategy.
- Farming was performed in a temperate climatic zone, and *Cyprinus carpio* GIT samples were monitored through a 6-week fish meat production cycle. This approach allows us to decipher the symbiotic microbiome of 33- to 37-week-old healthy carp, and we observed notable correlations between the fish GIT microbiota and the ABW.
- Marked differences were found in the distribution of the two dominant phyla: *Fusobacteria* and *Proteobacteria*. We did not find any significant enhancement in animal growth but detected strong positive correlations between the ABW and the relative frequencies of p__*Proteobacteria*, p__*Tenericutes*, c__*Mollicutes*, o__*Vibrionales*, f__*Peptostreptococcaceae*, f__*Pseudoalteromonadaceae* and f__*Lactobacillaceae*.
- No profound differences in the alpha and beta diversities were detected among the treatment groups, although anthocyanins (ANTH) and the pepper seed (fOS) and pulp (CAR) extracts were found to be promising for improving diversity.
- We did not observe any marked shifts in the abundance of opportunistic pathogen and spoilage genera such as *Shewanella*, *Pseudomonas* and *Aeromonas*. However, we noted that synbiotics (SYN) and fermentable oligosaccharides (fOS) decreased the relative proportions of *Aeromonas* and *Pseudomonas*, respectively, but the changes were not significant.

Phytonutrient-based feeding strategies might be a promising alternative for aquaculture systems because they exert a beneficial effect on the bacterial symbionts of the GIT of *Cyprinus carpio*.

## Supporting information

S1 Table Total amount of the raw materials used in the diets and nutrient contents of the experimental feeds (dry matter %). BD: basal diet (negative control), ANTH: BD+1% anthocyanins provided by sour cherry extract, SYN: BD+1% synbiotics provided by fermented corn, fOS: BD+1% fermentable oligosaccharides provided by sweet red pepper seed extract, CAR: BD+1% carotenoids provided by sweet red pepper pulp extract. *Vitamin and mineral premix: vitamin A (retinyl acetate), 9000000 IU; vitamin D3 (cholecalciferol), 7200000 IU; vitamin E, 5400 mg kg-1; vitamin K3 (MSB), 9600 mg kg-1; vitamin B1 (thiamin-HCL), 1000 mg kg-1; vitamin B2 (riboflavin), 9600 mg kg-1; vitamin B3 (niacin), 45000 mg kg-1; vitamin B5 (calcium d-pantothenate), 15000 mg kg-1; vitamin B6 (pyridoxine–HCL), 5400 mg kg-1; D-biotin, 100 mg kg-1; folic acid, 1200 mg kg-1; vitamin B12 (cyanocobalamin), 27 mg kg-1; vitamin C, 4000 mg kg-1; and choline chloride, 1500 mg kg-1. **Anchovy fish oil.
(PDF)

S1 Fig The UHPLC profiles of the anthocyanins and the identified main compounds, including the relative areas in ANTH. (Y axis: absorbance intensity (mAU); X axis: retention time (min)). The table shows the anthocyanin (ANTH) compounds of sour cherry with their retention areas and retention times.
(PDF)

S2 Fig The GC profile of the oligosaccharides and the identified monomer units with the greatest relative areas and retention times in SYN. (Y axis: counts; X axis: retention time (min)). The table shows the oligosaccharide monomers comprising the synbiotic (SYN) compounds of fermented corn with their relative retention areas and retention times.
(PDF)

S3 Fig The HPLC profile of the carotenoids and identified monomer units with the greatest relative areas and retention times in CAR. (Y axis: absorbance intensity (mAU); X axis: retention time (min)). Table identifies carotenoid compounds (CAR) of sweet red pepper pulp extract with relative percentage of areas and retention times.
(PDF)

S4 Fig The GC profiles of the oligosaccharides and the identified monomer units, including the greatest relative areas and retention times in fOS. (Y axis: counts; X axis: retention time (min)). Table identifies fermentable oligosaccharide monomers (fOS) of Hungarian sweet red pepper seed with relative percentage of areas and retention times.
(PDF)
