# Supplementary material for: Effects of phytonutrient-supplemented diets on the intestinal microbiota of Cyprinus carpio
Source: PLoS One. 2021 Apr 22;16(4):e0248537. doi: 10.1371/journal.pone.0248537 (PMC8062051; doi:10.1371/journal.pone.0248537)
Supplement: S1 Table — BD: basal diet (negative control), ANTH: BD+1% anthocyanins provided by sour cherry extract, SYN: BD+1% synbiotics provided by fermented corn, fOS: BD+1% fermentable oligosaccharides provided by sweet red pepper seed extract, CAR: BD+1% carotenoids provided by sweet red pepper pulp extract. *Vitamin and mineral premix: vitamin A (retinyl acetate), 9000000 IU; vitamin D3 (cholecalciferol), 7200000 IU; vitamin E, 5400 mg kg-1; vitamin K3 (MSB), 9600 mg kg-1; vitamin B1 (thiamin-HCL), 1000 mg kg-1; vitamin B2 (riboflavin), 9600 mg kg-1; vitamin B3 (niacin), 45000 mg kg-1; vitamin B5 (calcium d-pantothenate), 15000 mg kg-1; vitamin B6 (pyridoxine–HCL), 5400 mg kg-1; D-biotin, 100 mg kg-1; folic acid, 1200 mg kg-1; vitamin B12 (cyanocobalamin), 27 mg kg-1; vitamin C, 4000 mg kg-1; and choline chloride, 1500 mg kg-1. **Anchovy fish oil. (PDF) [file pone.0248537.s001.pdf]

Ingredients (g/100 g diet)	BD	ANTH	SYN	fOS	CAR
Poultry by-product meal	20	20	20	20	20
Blood meal (porcine haemoglobin)	2	2	2	2	2
JPC 56 soy protein concentrate	10	10	10	10	10
Fish meal, wild fish	15	15	15	15	15
Vitamin and mineral premix*	2	2	2	2	2
Zeolite	2	2	2	2	2
Glucose	1	1	1	1	1
Fish oil**	2	2	2	2	2
Experimental additive	0	1	1	1	1
Wheat meal	46	45	45	45	45
Total (100 g)	100	100	100	100	100

Analysed composition (dry matter basis)	BD	ANTH	SYN	fOS-s	fOS-p
Dry matter (DM)	90.26	90.39	90.39	90.39	90.39
Crude protein	33.5	33.39	33.39	33.39	33.39
Crude fat	6.91	6.90	6.90	6.90	6.90
Crude fibre	1.32	1.30	1.30	1.30	1.30
Ash	6.09	6.08	6.08	6.08	6.08
Digestible energy (MJ/kg)	15.04	14.92	14.92	14.92	14.92

S1 Table. Total amount of the raw materials used in the diets and nutrient contents of the experimental feeds (dry matter %). BD: basal diet (negative control), ANTH: BD+1% anthocyanins provided by sour cherry extract, SYN: BD+1% synbiotics provided by fermented corn, fOS: BD+1% fermentable oligosaccharides provided by sweet red pepper seed extract, CAR: BD+1% carotenoids provided by sweet red pepper pulp extract. *Vitamin and mineral premix: vitamin A (retinyl acetate), 9000000 IU; vitamin D3 (cholecalciferol), 7200000 IU; vitamin E, 5400 mg kg-1; vitamin K3 (MSB), 9600 mg kg-1; vitamin B1 (thiamin-HCL), 1000 mg kg-1; vitamin B2 (riboflavin), 9600 mg kg-1; vitamin B3 (niacin), 45000 mg kg-1; vitamin B5 (calcium d-pantothenate), 15000 mg kg-1; vitamin B6 (pyridoxine–HCL), 5400 mg kg-1; D-biotin, 100 mg kg-1; folic acid, 1200 mg kg-1; vitamin B12 (cyanocobalamin), 27 mg kg-1; vitamin C, 4000 mg kg-1; and choline chloride, 1500 mg kg-1. **Anchovy fish oil.
