# Supplementary material for: Effects of phytonutrient-supplemented diets on the intestinal microbiota of Cyprinus carpio
Source: PLoS One. 2021 Apr 22;16(4):e0248537. doi: 10.1371/journal.pone.0248537 (PMC8062051; doi:10.1371/journal.pone.0248537)
Supplement: S1 Fig — (Y axis: absorbance intensity (mAU); X axis: retention time (min)). The table shows the anthocyanin (ANTH) compounds of sour cherry with their retention areas and retention times. (PDF) [file pone.0248537.s002.pdf]

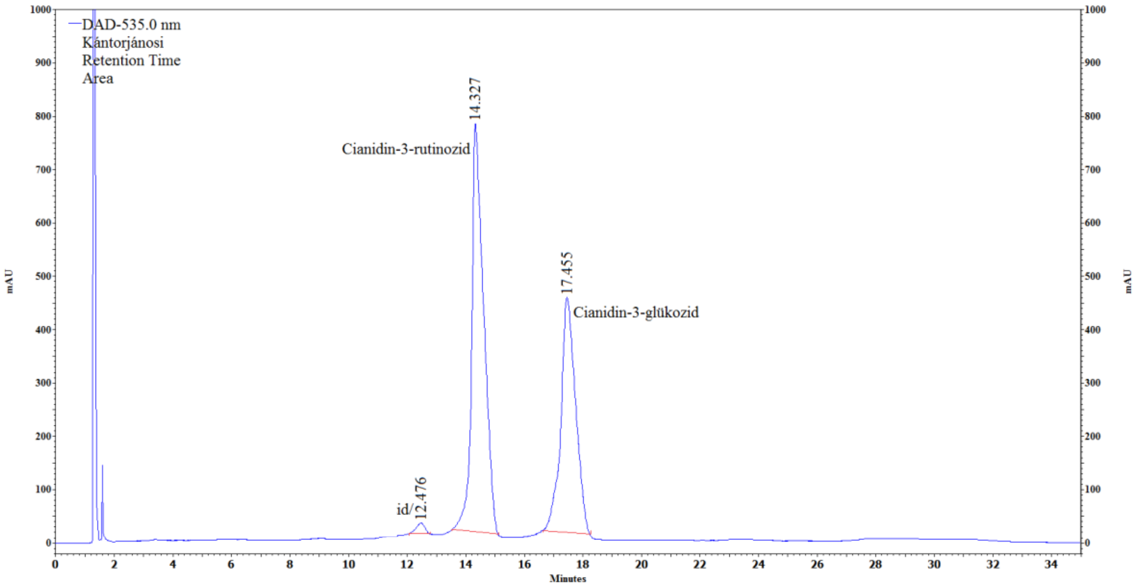

Anthocyanin compounds	Retention time (min)	Relative retention area (%)
Cyanidin-3-O-glucosyl-rutinoside	12.47	10.19
Cyanidin-3-O-rutinoside	14.32	54.61
Cyanidin-3-O-monoglucoside	17.45	35.19

S1 Fig. **The UHPLC profiles of the anhocyanins and the identified main compounds, including the relative areas in ANTH.** (Y axis: absorbance intensity (mAU); X axis: retention time (min)). The table shows the anthocyanin (ANTH) compounds of sour cherry with their retention areas and retention times.
