# Supplementary material for: Effects of phytonutrient-supplemented diets on the intestinal microbiota of Cyprinus carpio
Source: PLoS One. 2021 Apr 22;16(4):e0248537. doi: 10.1371/journal.pone.0248537 (PMC8062051; doi:10.1371/journal.pone.0248537)
Supplement: S2 Fig — (Y axis: counts; X axis: retention time (min)). The table shows the oligosaccharide monomers comprising the synbiotic (SYN) compounds of fermented corn with their relative retention areas and retention times. (PDF) [file pone.0248537.s003.pdf]

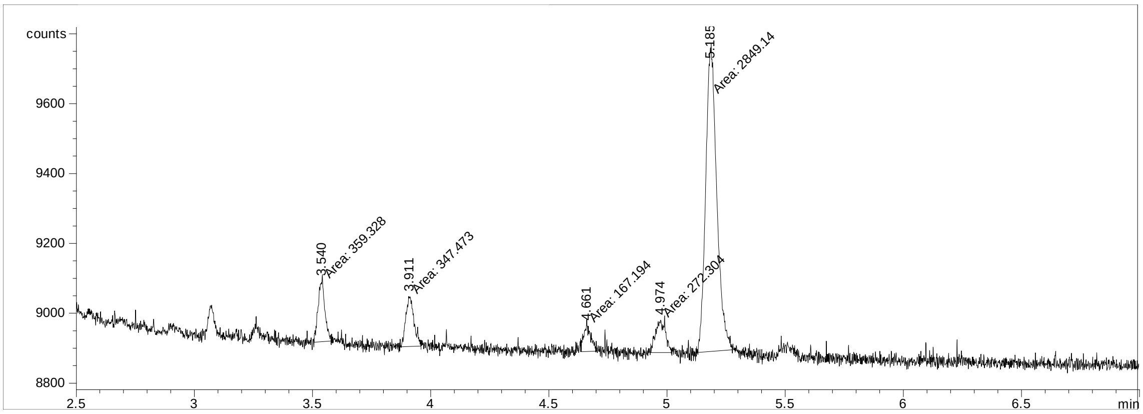

**Oligosaccharide monomers**	**Retention time (min)**	**Relative retention area (%)**
Arabinose	3.54	8.993
Xylose	3.911	8.697
Mannose	4.661	4.185
Galactose	4.974	6.815
Mannose	5.185	71.31

S2 Fig. **The GC profile of the oligosaccharides and the identified monomer units with the greatest relative areas and retention times in SYN.** (Y axis: counts; X axis: retention time (min)). The table shows the oligosaccharide monomers comprising the synbiotic (SYN) compounds of fermented corn with their relative retention areas and retention times.
