# Supplementary material for: Effects of phytonutrient-supplemented diets on the intestinal microbiota of Cyprinus carpio
Source: PLoS One. 2021 Apr 22;16(4):e0248537. doi: 10.1371/journal.pone.0248537 (PMC8062051; doi:10.1371/journal.pone.0248537)
Supplement: S4 Fig — (Y axis: counts; X axis: retention time (min)). Table identifies fermentable oligosaccharide monomers (fOS) of Hungarian sweet red pepper seed with relative percentage of areas and retention times. (PDF) [file pone.0248537.s005.pdf]

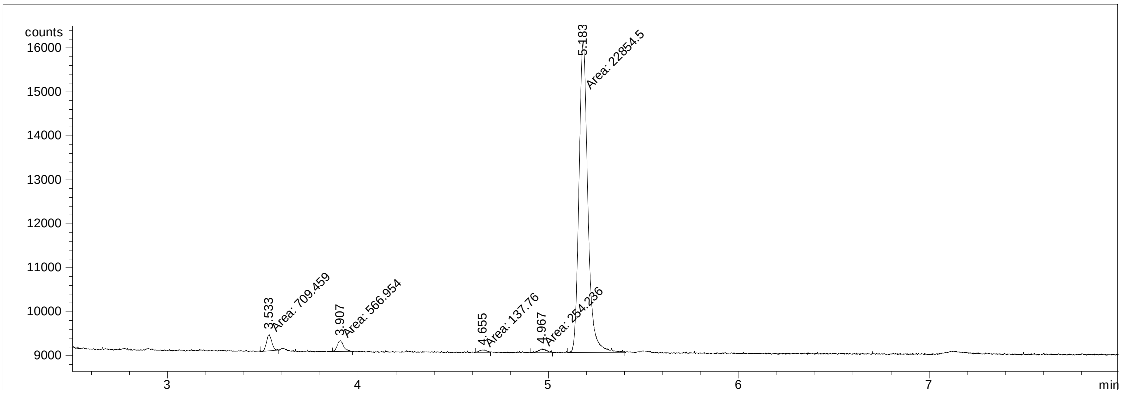

Name of oligosaccharide monomers	Retention time (min)	Relative area percentage (%)
Arabinose	3.533	2.893
Xylose	3.907	2.312
Mannose	4.655	0.562
Galactose	4.967	1.037
Mannose	5.183	93.197

S4 Fig. **The GC profiles of the oligosaccharides and the identified monomer units, including the greatest relative areas and retention times in fOS.** (Y axis: counts; X axis: retention time (min)). Table identifies fermentable oligosaccharide monomers (fOS) of Hungarian sweet red pepper seed with relative percentage of areas and retention times.
