# Supplementary material for: Effects of phytonutrient-supplemented diets on the intestinal microbiota of Cyprinus carpio
Source: PLoS One. 2021 Apr 22;16(4):e0248537. doi: 10.1371/journal.pone.0248537 (PMC8062051; doi:10.1371/journal.pone.0248537)
Supplement: S3 Fig — (Y axis: absorbance intensity (mAU); X axis: retention time (min)). Table identifies carotenoid compounds (CAR) of sweet red pepper pulp extract with relative percentage of areas and retention times. (PDF) [file pone.0248537.s004.pdf]

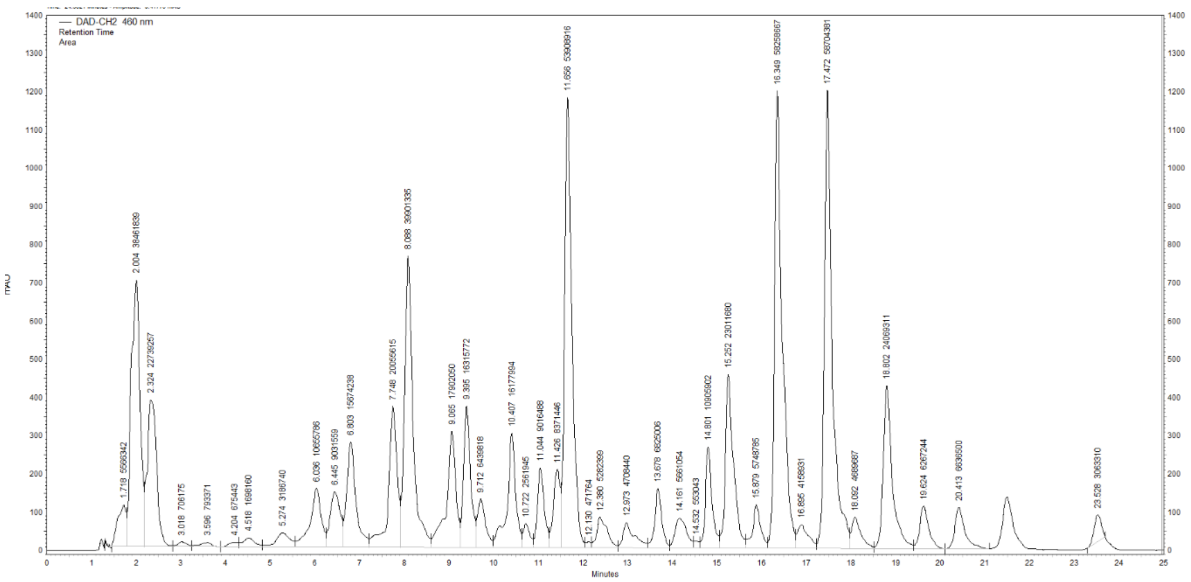

Carotenoid compounds	Retention time (min)	Relative retention area (%)
β-Carotene	11.656	9.67
cis-Capsanthin	16.349	10.44
Capsanthin	17.472	10.54
Zeaxanthin	18.802	4.39

S3 Fig. **The HPLC profile of the carotenoids and identified monomer units with the greatest relative areas and retention times in CAR**. (Y axis: absorbance intensity (mAU); X axis: retention time (min)). Table identifies carotenoid compounds (CAR) of sweet red pepper pulp extract with relative percentage of areas and retention times.
